# Supplementary material for: AffyMAPSDetector: a software tool to characterize Affymetrix GeneChip™ expression arrays with respect to SNPs
Source: BMC Bioinformatics. 2007 Jul 30;8:276. doi: 10.1186/1471-2105-8-276 (PMC1959249; doi:10.1186/1471-2105-8-276)
Supplement: Additional file 13 — AffyMAPSDetector v1 source code. AffyMAPSDetector source code. [file 1471-2105-8-276-S13.zip › AffyMAPSDetector_v1_src/doc/affychip/snpdetector/AffyMapsDetectorLauncher.html]

AffyMapsDetectorLauncher


|  |  |  |  |
| --- | --- | --- | --- |
| **Overview** \| **Package** \| **Class** \| **Use** \| **Tree** \| **Deprecated** \| **Index** \| **Help** | | |  |
| PREV CLASS   **NEXT CLASS** | **FRAMES**    **NO FRAMES**     **All Classes** |  |  |
| SUMMARY: NESTED \| FIELD \| CONSTR \| METHOD | DETAIL: FIELD \| CONSTR \| METHOD |  |  |


---


## affychip.snpdetector Class AffyMapsDetectorLauncher

```
java.lang.Object
  affychip.snpdetector.AffyMapsDetectorLauncher
```

---

public class **AffyMapsDetectorLauncher** extends java.lang.Object

Title: Probe SNP Detector

Description: Affymetrix Gene-Probe SNP Detection

Copyright: Copyright (c) 2004

Company: George Mason University

**Version:**
:   1.1

**Author:**
:   Sunita Kumari

---

**Constructor Summary**

`AffyMapsDetectorLauncher()`
  
          This is default contructor to create AffyMapsDetectorLauncher.


|  |  |
| --- | --- |
| **Method Summary** | |
| `static void` | `main(java.lang.String[] args)`             The main entry point into the application. |

**Methods inherited from class java.lang.Object**

`clone, equals, finalize, getClass, hashCode, notify, notifyAll, toString, wait, wait, wait`

**Constructor Detail**

### AffyMapsDetectorLauncher

```
public AffyMapsDetectorLauncher()
```

:   This is default contructor to create AffyMapsDetectorLauncher.


**Method Detail**

### main

```
public static void main(java.lang.String[] args)
```

:   The main entry point into the application. It installs platform specific
    look and feel besides bootstrapping the application.

    :   **Parameters:**: `args` - -- No arguments are needed to start AffyMapsDetector.


---


|  |  |  |  |
| --- | --- | --- | --- |
| **Overview** \| **Package** \| **Class** \| **Use** \| **Tree** \| **Deprecated** \| **Index** \| **Help** | | |  |
| PREV CLASS   **NEXT CLASS** | **FRAMES**    **NO FRAMES**     **All Classes** |  |  |
| SUMMARY: NESTED \| FIELD \| CONSTR \| METHOD | DETAIL: FIELD \| CONSTR \| METHOD |  |  |


---
